# Attenuation of Soft-Tissue Sarcomas Resistance to the Cytotoxic Action of TNF-α by Restoring p53 Function

**DOI:** 10.1371/journal.pone.0038808

**Published:** 2012-06-18

**Authors:** Jane Muret, Meriem Hasmim, Izabela Stasik, Abdelali Jalil, Aude Mallavialle, Arash Nanbakhsh, Ludovic Lacroix, Katy Billot, Véronique Baud, Jérome Thiery, Philippe Vielh, Philippe Terrier, Joelle Wiels, Lyubomir Vassilev, Axel Lecesne, Sylvie Bonvalot, Salem Chouaib

**Affiliations:** 1 Unité INSERM U753, Institut de Cancérologie Gustave Roussy, Villejuif, France; 2 Département d’Anesthésie, Institut de Cancérologie Gustave Roussy, Villejuif, France; 3 Unité INSERM U576, Groupe Hospitalier Archet, Nice, France; 4 Laboratoire de Recherche Translationnelle, Institut de Cancérologie Gustave Roussy, Villejuif, France; 5 Unité INSERM U1016, Institut Cochin, Paris, France; 6 CNRS, UMR 8104, Paris, France; 7 Université Paris Descartes, Sorbonne Paris Cité, Paris, France; 8 Département de Pathologie, Institut de Cancérologie Gustave Roussy, Villejuif, France; 9 UMR 8126, CNRS-IGR-Université Paris-XI, Institut de Cancérologie Gustave Roussy, Villejuif, France; 10 Discovery Oncology, Roche Research Center, Hoffmann-La Roche Inc., Nutley, New Jersey, United States of America; 11 Département de Médecine, Institut de Cancérologie Gustave Roussy, Villejuif, France; 12 Département de Chirurgie, Institut de Cancérologie Gustave Roussy (IGR), Villejuif, France; Vanderbilt University Medical Center, United States of America

## Abstract

**Background:**

Isolated limb perfusion with TNF-α and melphalan is used with remarkable efficiency to treat unresectable limb sarcomas. Here we tested the ability of TNF-α to directly induce apoptosis of sarcoma cells. In addition, we investigated the impact of p53 in the regulation of such effect.

**Methodology/Principal Findings:**

We first analysed the ability of TNF-α to induce apoptosis in freshly isolated tumour cells. For this purpose, sarcoma tumours (n = 8) treated *ex vivo* with TNF-α were processed for TUNEL staining. It revealed substantial endothelial cell apoptosis and levels of tumour cell apoptosis that varied from low to high. In order to investigate the role of p53 in TNF-α-induced cell death, human sarcoma cell lines (n = 9) with different TP53 and MDM2 status were studied for their sensitivity to TNF-α. *TP53^Wt^* cell lines were sensitive to TNF-α unless MDM2 was over-expressed. However, *TP53^Mut^* and *TP53^Null^* cell lines were resistant. TP53 suppression in *TP53^Wt^* cell lines abrogated TNF-α sensitivity and TP53 overexpression in *TP53^Null^* cell lines restored it. The use of small molecules that restore p53 activity, such as CP-31398 or Nutlin-3a, in association with TNF-α, potentiated the cell death of respectively *TP53^Mut^* and *TP53^Wt^*/*MDM2^Ampl^*. In particular, CP-31398 was able to induce p53 as well as some of its apoptotic target genes in *TP53^Mut^* cells. In *TP53^Wt^*/*MDM2^Ampl^* cells, Nutlin-3a effects were associated with a decrease of TNF-α-induced NF-κB-DNA binding and correlated with a differential regulation of pro- and anti-apoptotic genes such as TP53BP2, GADD45, TGF-β1 and FAIM.

**Conclusion/Significance:**

More effective therapeutic approaches are critically needed for the treatment of unresectable limb sarcomas. Our results show that restoring p53 activity in sarcoma cells correlated with increased sensitivity to TNF-α, suggesting that this strategy may be an important determinant of TNF-α-based sarcomas treatment.

## Introduction

Sarcomas are rare, poorly understood cancers, refractory to standard therapies [1], [2]. In this context, isolated limb perfusion (ILP), a highly specialized surgical technique that allows higher drug concentrations to be delivered loco-regionally, thus limiting treatment toxicity, can be used to treat unresectable soft tissue sarcomas (STS) of extremities avoiding limb amputation. During ILP, the treatment agents are circulated through the vasculature of the extremity using an extracorporeal pump oxygenator bypass circuit [3]. The combination of TNF-α and melphalan through ILP has been widely used to treat STS with impressive clinical responses [4], [5].

While the effect of melphalan, as a DNA-damaging alkylating agent, is clearly established, the effect of TNF-α remains controversial even though a TNF-α selective anti-vascular mechanism has been established [6]. Indeed, radiological studies demonstrated the selective disappearance of tumour hypervascularized areas after treatment with TNF-α [7]. Moreover, the tumour vascular disruption correlates *in vivo* with the induction of endothelial cell apoptosis [8] and *in vitro* with the specific suppression of αVβ3-mediated endothelial cell adhesion [9]. More recently, it has been demonstrated that VE-cadherin is a target of TNF-α, leading to the alteration of vascular integrity and tumour viability [10]. It has also been demonstrated that TNF-α increases intratumoural vessel permeability and reduces interstitial pressure, facilitating drug penetration in tumours [11]. These data suggest that the potent antitumour activity of ILP with TNF-α and melphalan is due to a dual targeting effect: first, TNF-α increases the permeability of tumour vessels and then melphalan reaches the tumour cells and induces cell death. However, a more complex process, in which TNF-α directly affects tumour cells, has also been postulated [12], [13].

TNF-α has been demonstrated to be either anti-proliferative, growth enhancing, or ineffective on transformed cell lines [14] as well as in early clinical trials when administered systemically [15]. In addition to this wide variation in sensitivity of tumour cells, the therapeutic value of TNF-α in the treatment of cancer has been limited by toxicity at high doses [3]. Even if the locoregional administration of TNF-α by ILP is able to partially attenuate its systemic toxic side effects, the resistance of some tumour cells to the cytotoxic action of TNF-α remains a barrier to its effective application [16]. Therefore, understanding the molecular and biochemical mechanisms of tumour cell resistance to the cytotoxic action of TNF-α may ultimately provide new approaches to enhance the therapeutic effectiveness of TNF-α against human malignancies [17].

In this regard, the dependence of TNF-α-induced cell death on *TP53* status was reported *in vitro* [18], [19] and *in vivo* in sarcoma patients treated with TNF-α and melphalan [20]. It should be noted that *TP53* is mutated in more than 20% or non functional in up to 60% of STS (IARC *TP53* mutation database, R15 release, Nov. 2010) [21]. Moreover, a recent study on 143 various STS showed that the p53/p14 pathway was altered in all analysed tumours [22]. Furthermore, it is well established that p53 may retain its wild-type configuration while being functionally repressed through epistatic mechanisms, most frequently via over-expression of MDM2 protein and *MDM2* gene amplification (*MDM2^Ampl^*). Importantly, *TP53* mutations or inactivations were found to correlate with a poorer outcome in sarcoma patients [23].

It has been reported that pharmacological and/or genetic restoration of the wild-type p53 pathway induce tumour cell death *in vitro* and inhibit tumour growth in animal models [24]. The recent emergence of small molecules restoring the wild-type conformation of mutant p53 protein, as well as blocking the MDM2-p53 interaction, is likely to become a promising anti-tumour strategy. In this context, the conformational changes observed in the case of missense mutation of p53 are reversible at least to some extent. Thus, CP-31398, a styrylquinazoline, can rescue some mutant p53 to a wild-type conformation and therefore increase the steady-state wild-type p53 to high levels equivalent to those observed following DNA damage [25], [26]. Nutlins are imidazoline derivatives that specifically bind and dissociate MDM2 from p53, thereby rescuing p53 from degradation [27]. Thus, such molecules have been reported to efficiently re-activate p53, resulting in cell-cycle arrest and apoptosis [28], [29].

In the present study, we demonstrate that TNF-α induces cell death not only in tumour vasculature but also in sarcoma tumour cells harboring wild type p53, and that resistance to TNF-α could be linked to lack of p53 function in this type of tumour. Furthermore, we demonstrate that combining TNF-α with drugs that restore the p53 pathway enhances global apoptotic effects. These findings suggest that this combination therapy approach could be used to treat highly resistant sarcoma tumours.

## Methods

### Ethics Statement

The Institutional Review Board of the Gustave Roussy Institute (Commission Scientifique des Essais Thérapeutiques) specifically approved the study and all patients included in the study signed an informed consent.

### Antibodies and Reagents

Mouse anti-CD31 monoclonal antibody conjugated with Alexa Fluor 488 was purchased from BD Pharmingen; mouse anti-p53 (DO-1) and anti-TNFR1 antibodies from Santa Cruz Biotechnology (Santa Cruz, Ca, USA); anti-p21 and anti-MDM2 from Calbiochem (Darmstadt, Germany); rabbit anti-BAX polyclonal antibodies from Cell Signaling Technology (Danvers, MA, USA); mouse anti-GAPDH monoclonal antibody from Millipore (Billerica, MA, USA) and mouse anti-actin monoclonal antibody from Sigma-Aldrich (St Louis, MO, USA). Human recombinant TNF-α (Beromun) was obtained from Boehringer Ingelheim (Ingelheim am Rhein, Germany). Nutlin-3a was kindly provided by Dr L. Vassilev (Hoffman La Roche, Nutley, USA) and CP-31398 by Dr L. Kopelovich (NIH/NCI, Bethesda, USA). TO-PRO3 was purchased from Invitrogen (Carlsbad, CA, USA).

### *In Situ* Cell Death Detection in Fresh STS Human Tumours

Pieces of fresh sarcoma tumours were obtained after the surgical treatment of eight patients with resectable STS of the retroperitoneum. Tumours were immediately embedded in 6% low-melting point agarose (Sigma, St. Louis, MO, USA). 200 µm thick slices were cut at room temperature with Leica VT 1000 S Microtome (Leica Microsystems, Wetzlar, Germany). The slices were incubated in culture medium (RPMI containing 10% FCS) with or without 50 ng/ml of TNF-α for 90 min at 37°C and 5% CO_2_, then washed and maintained in the same conditions for 72 h in fresh culture medium without TNF-α. Successively, cultured slices were washed in PBS and fixed by immersion in cold methanol for 10 min at −20°C. TUNEL reaction was performed using *in situ* Cell Death Detection Kit (Roche, Foster City, CA, USA) according to manufacturer’s instructions. Next, the slices were incubated overnight at 4°C with mouse anti-CD31 antibody conjugated with Alexa Fluor 488 and subsequently for 1 h with TO-PRO3. Slices were finally mounted on glass slides using fluoromount-G (SouthernBiotech, Birmingham, UK). Stacks of confocal slices were collected with a LSM 510 laser scanning confocal microscope (Carl Zeiss, Jena, Germany) using 20X/0.75 Plan-Apochromat objective. The excitation wavelengths were 488 nm for Alexa 488, 543 nm for the TUNEL positive nuclei revealed by the Cell Death Detection Kit, and 633 nm for TO-PRO3. Images were acquired using BP505–530, BP560–615 and LP 650 emission filters respectively. Z-projection of stacks was done using LSM Image Examiner software (Zeiss, Le Pecq, France). Cell counting was done by ImageJ software using the cell counter plugin and the percentage of apoptotic endothelial and tumour cells was determined.

### Cell Lines

Rhabdomyosarcoma cell line (KYM-1) [30] was kindly provided by Dr. T. Meager (National Institute for Biological Standards and Control, United Kingdom), fibrosarcoma cell line (HT1080) [31] by Dr. L. Maggiorella (IGR, Villejuf, France). Liposarcoma cell lines (T449, T778 and T1000) [32], [33] were provided by Dr. F. Pedeutour (Nice University, Nice, France) malignant fibrous histiocytoma (MFH152, MFH100, MFH95) [34] and leiomyosarcoma (LMS148) [35] cell lines were provided by Dr. A. Aurias (Institut Curie, Paris, France).

### Cell Culture and Treatments

HT1080 cells were grown in DMEM (4.5 g/ml glucose) with 10% FCS, and the remaining cell lines in RPMI supplemented with 10% FBS and 1% sodium pyruvate (Gibco, Villebon sur Yvette, France). To study TNF-α effects, cells were seeded at 25% confluency, and one day later they were either treated with 50 ng/ml TNF-α alone or in combination with 7.5 µg/ml CP-31398 or 5 µM Nutlin-3a for the indicated times. For cell irradiation, we administered 15 Gy using the MXR−225/22 X-ray machine (Comet AG, Flamett, Switzerland) at a dose rate of 1 Gy/min.

### *TP53* Sequencing

DNA from cell lines was extracted from cell pellets with QIAamp Blood Tissue Kit (Qiagen, Hilden, Germany) according to manufacturer’s instructions. Resequencing of *TP53* gene was performed by Sanger sequencing based on PCR product and did not include generation of new data as the reference sequence used for primer design and for alignment was already published and available in RefSeq database as NM_000546.4 for *TP53* (tumor protein p53; Other Aliases: FLJ92943, LFS1, TRP53, p53). PCR amplification of 9 amplicons covering exons 2 to 11 (corresponding to the whole coding sequence), including at least 20 bases in flanking introns was performed. Detailed protocol and primer sequences are available on demand. Purified DNA was sequenced using BigDye® Terminator Cycle Sequencing Kit (Applied Biosystems, Foster City, CA, USA). Sequencing reactions were analyzed on 48-capillary 3730 DNA Analyzer® in both sense and antisense directions. All mutations detected were controlled with independent amplification at least one time. Sequence reading and alignment were performed with SeqScape® software (Applied Biosystems, Foster City, CA, USA).

### Apoptosis Detection

Cell death was assessed by 3.3-dihexyloxacarbocyanine iodide (DiOC_6_) and propidium iodide labeling (Molecular Probes, Carlsbad, CA, USA) after 72 h treatment with TNF-α alone or in combination with CP-31398 or Nutlin-3a. The samples were analyzed on a FACSCalibur flow cytometer and data were processed using CellQuest software (Becton Dickinson, Franklin Lakes, NJ USA).

### Western Blot Analysis

Western blot analyses were performed using specific monoclonal or polyclonal antibodies according to standard protocols. Briefly, washed cells were lysed in an adherent state in a lysis buffer containing 3.75% SDS, 15% glycerol, 0.3 M Tris-HCl (pH6.8), and a protease inhibitor mixture (Complete Protease Inhibitor Mixture, Roche Molecular Biochemicals, Foster City, CA, USA). Equivalent protein extracts (30 or 50 µg) were resolved by SDS–PAGE, transferred to a nitrocellulose membrane (Millipore, Billerica, MA, USA), and incubated overnight with appropriate primary antibodies at 4°C. The labeling was visualized using horseradish peroxidase-conjugated secondary antibodies and by enhanced chemiluminescence (ECL Western blotting Kit, Amersham Biosciences, Vélizy, France).

### Flow Cytometry

Cells were harvested using EDTA 1 mM, washed twice with PBS, and incubated with anti-TNFR1 or isotype control antibodies (Santa Cruz Biotechnology, Santa Cruz, CA, USA) for 30 min. at 4°C, followed by incubation with phycoerythrin-conjugated goat anti-mouse antibody (Beckman Coulter, Villepinte, France). The samples were analyzed on a FACSCalibur flow cytometer and data were processed using CellQuest software (Becton Dickinson, Franklin Lakes, NJ, USA).

### Transfections

HT1080 transfection was performed using Lipofectamine2000 Transfection Reagent (Invitrogen, Villebon sur Yvette, France), according to the manufacturer’s instructions. Expression vectors encoding shRNA designed for specific silencing of p53 or luciferase were purchased from InvivoGen. Stably transfected HT1080clones were selected with 500 µg/ml Zeocin (InvivoGen, Toulouse, France). MFH95 cells, MFH100, and MFH152 cells were transfected by electroporation in an Easyject electroporation system (Equibio, 260 V, 450 µF) using the p53 encoding plasmid pC53-SN3 for MFH-95.

### Cell Cycle Analysis

Harvested cells were gently fixed in 70% cold-ethanol, and incubated over night at −20°C. 70% ethanol was then removed by centrifugation, and after PBS-washing, pelleted cells were incubated at 37°C for 1 hour in a mixture containing 0.5 ml PBX1x, 10 µl of 10 mg/ml RNAse A, and 25 µl of 1 mg/ml propidium iodide solution. Cell cycle analysis was then performed by flow cytometry.

### Electrophoretic Mobility Shift Assay (EMSA)

For analysis of NF-κB DNA binding activity, 15 µg of nuclear extracts were incubated with the radioactive labelled HIV-LTR tandem κB oligonucleotide as κB probe [36]. The samples were separated on a non-denaturating PAGE gel and signals were detected by autoradiography.

### Real-time Quantitative PCR Analysis

For apoptotic gene expression analysis, total RNA was extracted after 16 h of treatment using Trizol (Sigma-Aldrich, St. Louis, MO, USA) following manufacturer’s instructions, and reverse transcription was performed using a High Capacity cDNA Reverse Transcription Kit (Ambion, Foster City, CA, USA). For quantification of apoptosis related genes, real time PCR (RT-PCR) was performed using Platinum SYBR green qPCR SuperMix-UDG w/ROX (Invitrogen, Villebon sur Yvette, France) and the 7900 HT Fast Real-time PCR System (Applied Biosystems, Foster City, CA, USA). Apoptosis dedicated arrays were elaborated by the PETC platform of INSERM U576 and INSERM U876. The relative expression level of 91 apoptosis-related genes was normalized using four different house-keeping genes (GADPH, β-actin, HPRt and PPIA). For each sample, cycle threshold (Ct) values for the housekeeping genes were determined for normalization purposes, and delta Ct (ΔCt) between the mean of target-gene values and housekeeping-genes values was calculated. Relative expression levels of target gene mRNAs between control cells and Nutlin-3a or Nutlin-3a + TNF-α-treated cells were calculated and expressed as fold change over control. Values represent the mean of duplicates and are representative of two independent experiments.

### Statistical Analysis

Results are expressed as mean ± standard deviation (SD) of values obtained in at least three independent experiments. Differences between samples were analysed using the 1- way ANOVA. When it was statistically significant, post-hoc analysis used Student t-test with Bonferroni corrections. Differences were considered statistically significant for p value inferior to 0.05/number of comparisons. All calculations were performed using the Microsoft Office Excel statistical analysis software 2003.

## Results

### TNF-α Induces Apoptosis of Sarcoma Tumour Cells During *Ex Vivo* ILP

**Figure 1:**
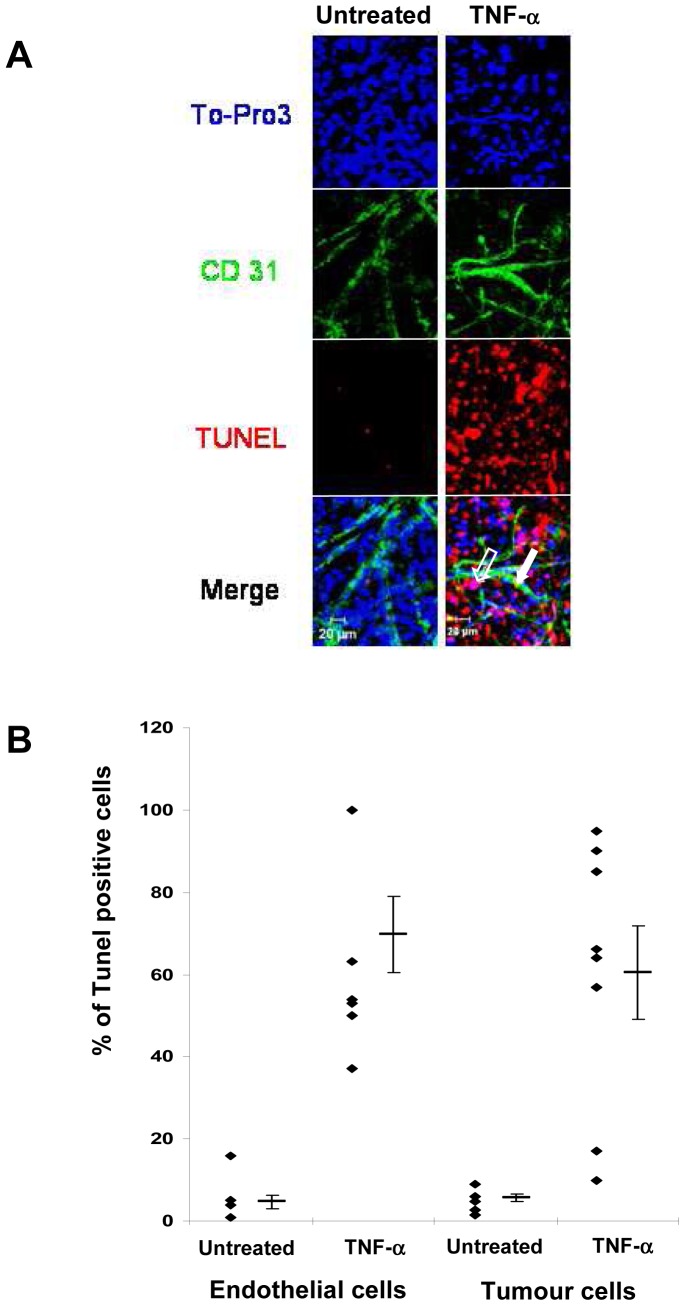
TNF-α induces apoptosis of sarcoma cells. *A*, Confocal microscopy analysis of *in situ* cell death experiments. Slices from fresh tumors maintained in culture with or without TNF-α were processed for TO-PRO3 staining (blue), Alexa Fluor 488 conjugated mouse anti-CD31 antibody (green), and TUNEL assay (red). Untreated slices are represented on the left panel and TNF-α-treated slices on the right. Apoptotic tumor cells appear purple (empty arrow) in the merged images at the bottom. Apoptotic endothelial cells that have undergone apoptosis appear yellow (solid arrow). *B,* Quantification of TUNEL-positive cells. Apoptotic cells were counted among 500 cells for each tumour in untreated as well as in TNF-α treated samples. Results are expressed in percentage of TUNEL-positive cells. Results are expressed as mean ± SEM.

Although it is postulated that TNF-α works via its selective antivascular mechanisms, its contribution to the antitumour effect of ILP is still to be elucidated. In order to determine the antitumour effects of TNF-α, an *ex vivo* tumour culture assay, recapitulating conditions that might occur *in vivo* during ILP, was performed. Slices from eight retroperitoneal sarcomas prepared immediately after surgery (well differentiated liposarcoma, n = 4; undifferentiated sarcoma, n = 2; and leiomyosarcoma, n = 2) were treated with or without TNF-α for 90 min, washed and then maintained in fresh medium without TNF-α for 72 h before TUNEL assay and subsequent labelling with anti-CD31 antibody and TO-PRO3. Double staining of endothelial cells with anti-CD31 antibody (green) and TUNEL (red) showed co-localization of both colours (yellow), indicating that TNF-α induces apoptosis of endothelial cells. Moreover, co-localization of nuclear staining (TO-PRO3, blue) and TUNEL (red), resulting in purple, also indicates that TNF-α induces apoptosis of tumour cells. Untreated slices showed no or few TUNEL-positive nuclei, excluding the possibility that spontaneous apoptosis was induced by our experimental conditions (Fig. 1A). Further quantification of the TUNEL-positive cells also revealed that TNF-α induces apoptosis of endothelial cells in most patients (69.6±9%) whereas for tumour cells, patients could be subdivided into three groups: patients with low apoptosis rate (10% to 17%, n = 2), patients with moderate apoptosis rate (57 to 66%, n = 3), and patients with high apoptosis rate (85% to 95%, n = 3), suggesting that sarcoma tumour cells are differentially sensitive to TNF-α-induced apoptosis (Fig. 1B).

### Mutation Status of TP53 and Impact on TNF-α-induced Cell Death in Sarcoma Cell Lines

**Figure 2:**
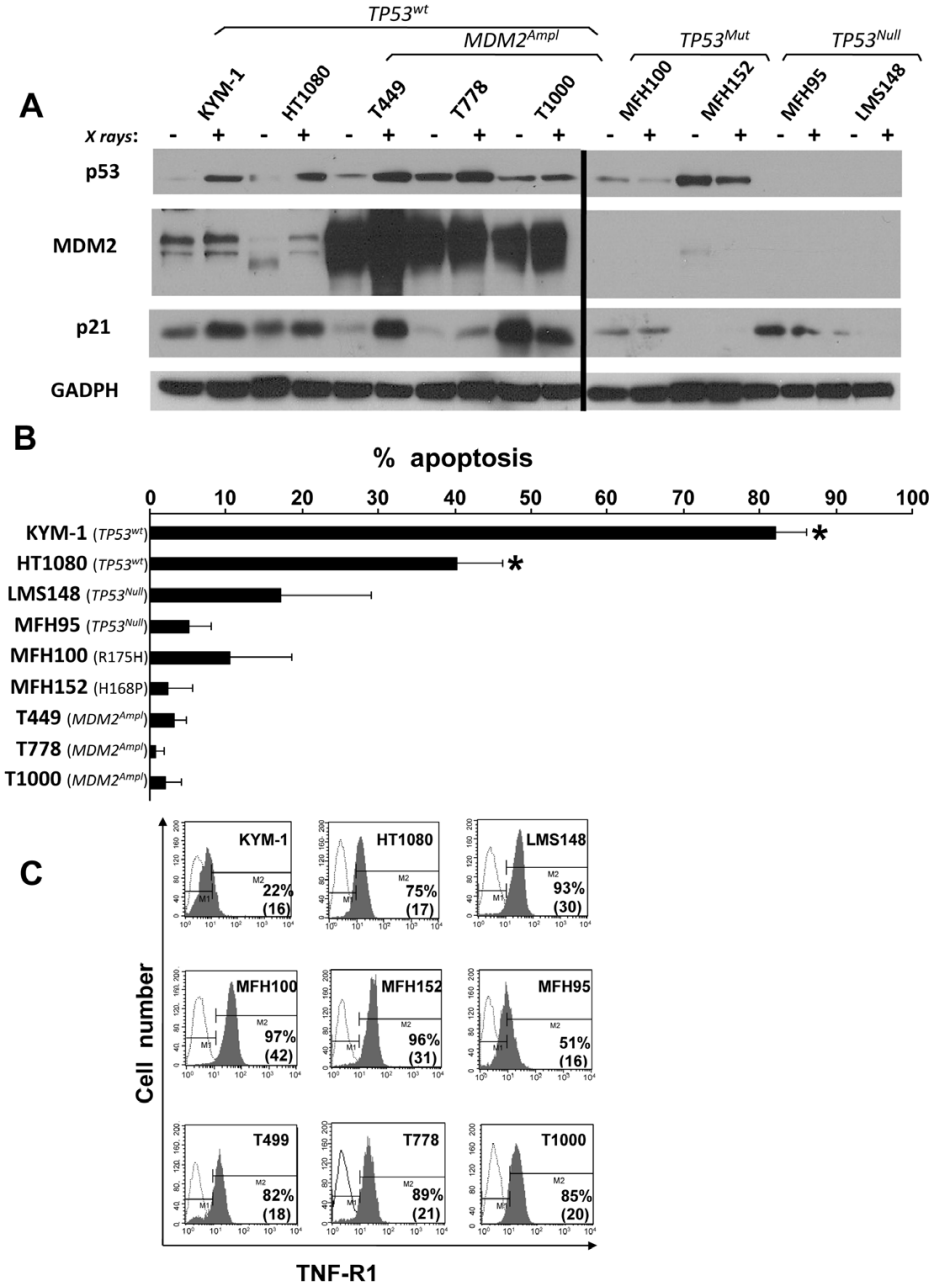
TNF-α induced cell death in nine sarcoma cell lines with different p53 status. *A,* Western blot analysis of p53, MDM2 and p21 expression in *TP53^Wt^*/*MDM2^Wt^* (KYM-1 and HT1080), *TP53^Wt^*/*MDM2^Ampl^* (T449, T778 and T1000), *TP53^Mut^* (MFH100 and MFH152) and *TP53^Null^* (MFH95 and LMS148) cell lines studied after irradiation. Lysates were prepared 2 h after exposure to 15 Gy. *B*, Impact of TNF-α on cell survival assessed by DiOC_6_/PI method after 72 h treatment. The percentage of specific apoptosis is shown as an increase of apoptotic cells (early and late apoptosis) in comparison to untreated cells. Results from four experiments are shown. *p<0.05 *versus* all cell lines. *C,* Cell surface expression of TNF receptor type 1 detected by flow cytometry following staining with TNFR1-specific monoclonal antibody (filled histograms) and isotype-matched control (open histograms). Percentages of positive cells and of mean fluorescence intensity are indicated; representative results from three experiments are shown.

Among a selection of nine different sarcoma cell lines, the sequencing of full-length p53 cDNAs revealed that five cell lines (KYM-1, HT1080, T449, T778 and T1000) had wild type TP53 gene (*TP53^Wt^*). In MFH100 and MFH152 cells, *TP53* had respectively a homozygous 524 G>A (R175H) and 503 A>C (H168P) missense mutation (*TP53^Mut^*). In MFH95 and LMS148 cells, a *TP53* deletion was observed since no amplification of *TP53* exons 2 to 11 could be obtained (*TP53^Null^*). Moreover, Western blot analysis showed that T449, T778 and T1000 cells over-expressed MDM2 while MFH100, MFH152, MFH95 and LMS148 cells displayed non-functional p53 pathway based on the absence of p53 increase following irradiation (Fig. 2A).

As *TP53* status might modulate TNF-α-induced cell death efficiency, we analysed TNF-α-induced apoptosis on our panel of nine soft tissue sarcoma cell lines by 3.3-dihexyloxacarbocyanine iodide (DiOC_6_) and propidium iodide (PI) labeling after 72 h of TNF-α treatment. *TP53^Wt^*/*MDM2^Wt^* cell lines (KYM-1 and HT1080) displayed high levels of apoptotic cells after TNF-α treatment (40% and 82% respectively) (Fig. 2B) whereas *TP53^Null^, TP53^Mut^* and *TP53^Wt^*/*MDM2^Ampl^* cell lines were resistant to TNF-α-induced cell death (0.7% to 17% of apoptosis). To verify if the resistance to TNF-α was not due to a low expression level of the TNF receptor type 1 (TNF-R1), we assessed the surface expression of TNF-R1 by flow cytometry (Fig. 2C). Most of the cell lines expressed high levels of TNF-R1 (51% to 97%), regardless of p53 status, showing that resistance of STS cell lines to TNF-α-induced apoptosis was not due to the absence or low expression of TNF-R1. Taken together, these results demonstrate a high correlation between p53 pathway status (*TP53^Wt^* versus *TP53^Null^, TP53^Mut^* or *TP53^Wt^*/*MDM2^Ampl^*) and sensitivity to TNF-α-induced apoptosis.

### Level of Expression of p53 Influences TNF-α-induced Cell Death

**Figure 3:**
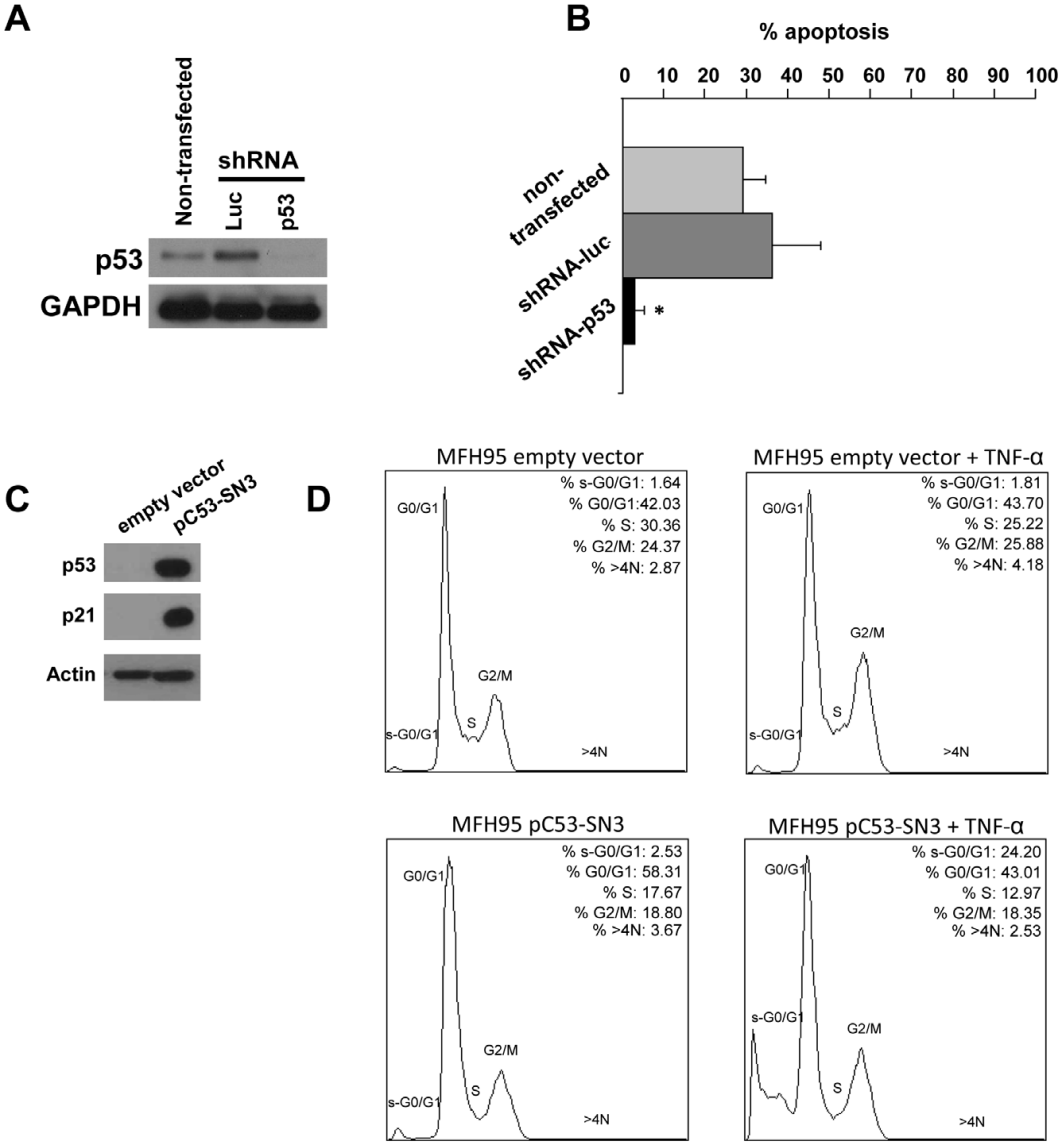
Effects of p53 silencing and overexpression on TNF-α-induced cell death. *A*, *TP53^Wt^*/*MDM2^Wt^* cell lines (HT1080) were transfected with expression vectors encoding shRNA specific for either luciferase (shRNA-luc) or p53 (shRNA–p53) and efficiency was controlled by Western-blot. *B,* Apoptosis induction after 72 h TNF-α treatment in non-transfected and transfected (shRNA) HT1080 cell lines. Representative results of induced apoptosis from two experiments are shown. *p<0.05 *versus* p53 expressing cells transfected and non-transfected by shRNA-Luc. *C*, MFH95 *TP53^Null^* cell lines were transfected with p53-encoding plasmid pC53-SN3 and efficiency was controlled by Western-blot. *D,* Cell cycle analysis of control and p53-expressing MFH95 cell lines treated or not by TNF-α. Percent of cells in each phase of the cell cycle is indicated at the upper right corner.

In order to confirm the role of p53 in TNF-α-induced apoptosis of STS, we first knocked down p53 in the *TP53^Wt^*/*MDM2^Wt^* TNF-α sensitive HT1080 cell line by stable transfection of a vector encoding a short hairpin RNA (shRNA) targeting p53 (shRNA-p53) or luciferase mRNA as a control (shRNA-luc) (Fig. 3A). Silencing of p53 resulted in the abrogation of TNF-α-induced apoptosis (29% and 36% apoptosis respectively in non-transfected and control cells versus 3% in shRNA-p53 cells, p<0.05) (Fig. 3B). Moreover, the inhibition of TNF-α-induced apoptosis in shRNA-p53 transfected cells was not due to the decrease of TNF-R1 expression (data not shown).

We also transient overexpressed p53 in *TP53^Null^* TNF-α resistant MFH95 cell line using pC53-SN3 expression plasmid. We obtained an efficient p53 expression associated with a concomitant induction of p21 (Fig. 3C). To investigate the effect of TNF-α on these cells, we performed a cell cycle analysis to evaluate a putative cell cycle arrest due to p21 induction and to quantify cell death represented by sub-G1 cells. We obtained an increase in G1-phase (from 42% to 58%) as well as a decrease in S-phase (from 30% to 17%) when p53 was overexpressed in MFH95 cells, suggesting a G1-arrest (Fig. 3D). In the presence of TNF-α there was no effect in control cells whereas the proportion of sub-G1 cells drastically increased in p53-expressing MFH95 cells (24%) compared to control cells (1.8%) and to untreated p53-expressing cell (2.5%). These results demonstrate that p53 expression in *TP53^Null^* MFH95 cells enhanced TNF-α-induced cell death.

Altogether, these data further highlight the role of p53 in regulating TNF-α-induced cell death in sarcoma cell lines.

### CP-31398 Sensitizes *TP53^Mut^* Sarcoma Cell Lines to TNF-α-induced Cell Death

**Figure 4:**
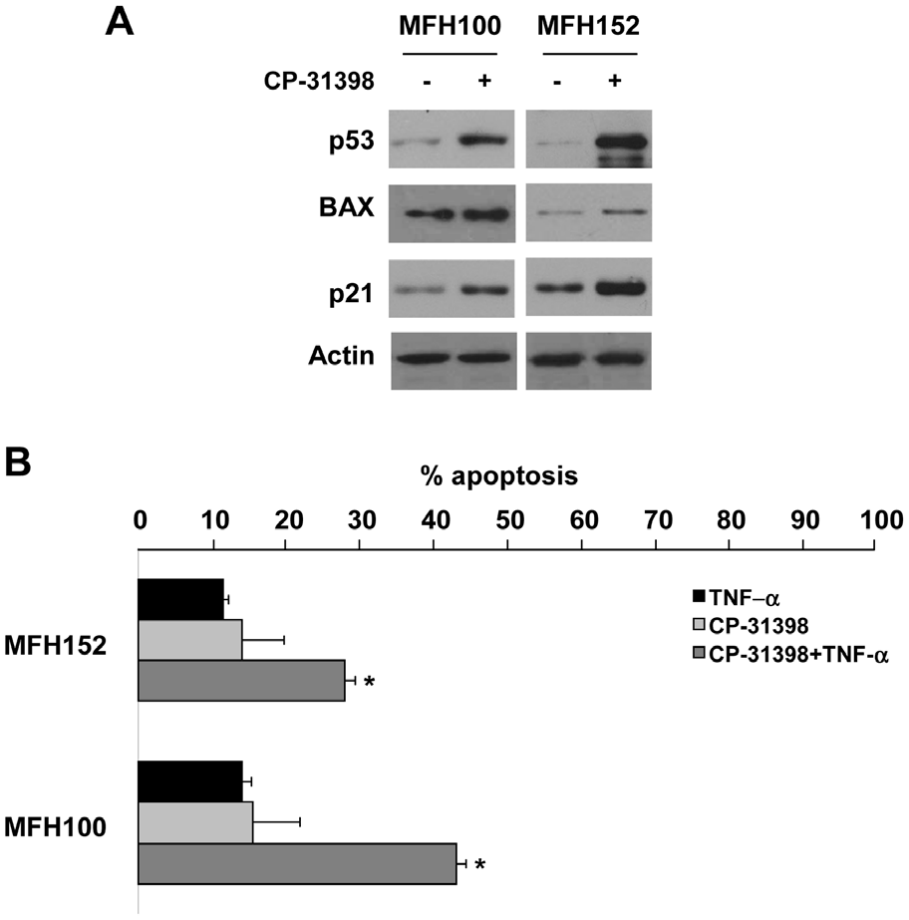
Restoration of p53 function with CP-31398 sensitizes sarcoma cells to TNF-α-induced apoptosis. *A,* Western-blot analysis of p53 target gene expression in *TP53^Mut^* cell lines (MFH100 and MFH152) after 24 h treatment with CP-31398. *B*, Apoptosis induction after 96 h of culture. Cells were treated either with CP-31398 alone (24 h), TNF-α alone (72 h) or both sequentially (24 h with CP followed by 72 h with TNF-α). Apoptosis was measured after 96 h by DiOC_6_/PI labeling; results from three independent experiments are shown. * p<0.05 versus CP-31398 or TNF-α treated cells.

The small molecule CP-31398 was reported to stabilize the wild-type-associated epitope (mAb1620) of the p53 DNA-binding domain, thus conferring a wild-type conformation to mutant p53 and rescuing p53 functions [37]. Therefore, we asked whether sarcoma cell lines treatment with CP-31398 would enhance p53 protein expression and its transcriptional activity. Western blot analysis showed an increase in p53 protein level in MFH100 and MFH152 *TP53^Mut^* cell lines after 24 h CP-31398 treatment (Fig. 4A). Furthermore, CP-31398 treatment increased expression of the p53 targets p21 and BAX. These results illustrate the efficacy of CP-31398 in restoring p53 functional activity in our p53-mutated STS cell lines. In order to investigate whether CP-31398 can restore the sensitivity of *TP53^Mut^* cell lines to TNF-α-induced cell death, we incubated MFH152 and MFH100 cells with 50 ng/ml TNF-α and/or CP-31398 (Fig. 4B). Results show that CP-31398 alone had a slight apoptotic effect. However, CP-31398 pre-treatment followed by 72 h TNF-α had a synergistic effect on apoptosis induction in both *TP53^Mut^* cell lines (28% and 43% respectively for MFH152 and MFH100). These results show that in *TP53^Mut^* cell lines, restoration of wild-type p53 activity can enhance susceptibility to TNF-α induced cell death.

### Nutlin-3a Sensitizes *TP53^Wt^/MDM2^Ampl^* Sarcoma Cell Lines to TNF-α Cytotoxic Action

**Figure 5:**
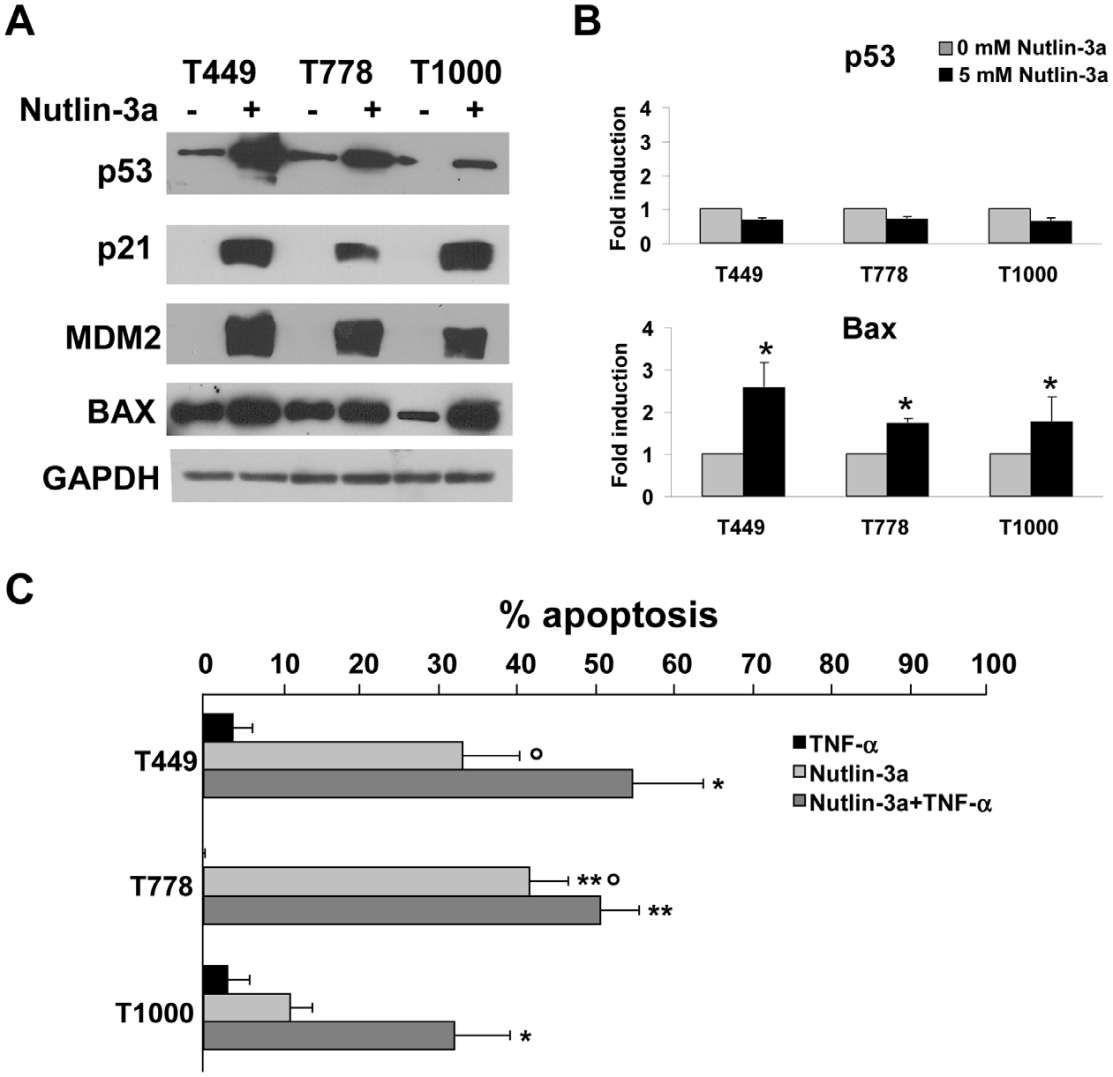
Restoration of p53 pathways by Nutlin-3a in TP53Wt/MDM2Ampl cells. A, Induction of p53 target genes in TP53Wt/MDM2Ampl (T449, T778 and T1000) cell lines after 16 h treatment with Nutlin-3a detected by Western blotting. B, Expression of p53 and BAX transcripts after 16 h treatment with Nutlin-3a detected by RT-PCR. The results are shown as fold induction relative to cells treated with medium. * p<0.05 versus untreated cells. C, Apoptosis induction after Nutlin-3a or/and TNF-α treatment for 72 h in TP53Wt/MDM2Ampl cell lines. Apoptosis was detected by DiOC6/PI assay. Results from three independent experiments are shown. * p<0.05 versus T449 or T1000 with TNF-α or Nutlin-3a treatment. p<0.05 versus T1000 with Nutlin-3a treatment. ** p<0.05 versus T778 with TNF-α treatment.

We then examined the effect of the MDM-2 inhibitor Nutlin-3a on TNF-α induced cell death. Nutlin-3a is a small-molecule antagonist of MDM2 that binds in the p53-binding pocket, preventing p53 degradation and thus restoring its transcriptional activity [27] and leading to cycle cell arrest and apoptosis. *TP53^Wt^*/*MDM2^Ampl^* sarcoma cell lines (T449, T778 and T1000) were incubated with Nutlin-3a for 16 h. Western blot analysis showed an increase of p53 protein level leading to p21 expression and an increase of BAX expression, suggesting a restoration of p53 transcriptional activity after treatment (Fig 5A). Similar results were also obtained by quantitative RT-PCR analysis, where the increase in p53 protein level was associated with an increase of BAX mRNA expression (Fig. 5B). However, the observed increase in p53 protein was not accompanied by an increase of the p53 mRNA (Fig. 5B), presumably due to post-transcriptional regulation by Nutlin-3a.

We then tested the effect of Nutlin-3a treatment alone or in combination with TNF-α on cell viability, as recent studies suggested synergistic effects of Nutlins with other chemotherapies [33]. 72 h treatment with Nutlin-3a induced cell death in T449 and T778 cell lines (33% and 41.5% respectively) but had only minor effects on T1000 cells (11%) (Fig. 5C). Incubation of cells with both TNF-α and Nutlin-3a for 72 h resulted in potentiation of TNF-α-induced cell death in T449 and T1000 cell lines (with a respective cell death increase to 54.7% and 32% compared to Nutlin-3a alone) (Fig. 5C). However, this potentiation was not observed in T778 cells displaying maximal cell death in the presence of Nutlin-3a.

### Inhibition of TNF-α-induced NF-κB Activity and Differential Apoptotic Gene Regulation in the Presence of Nutlin-3a

**Figure 6:**
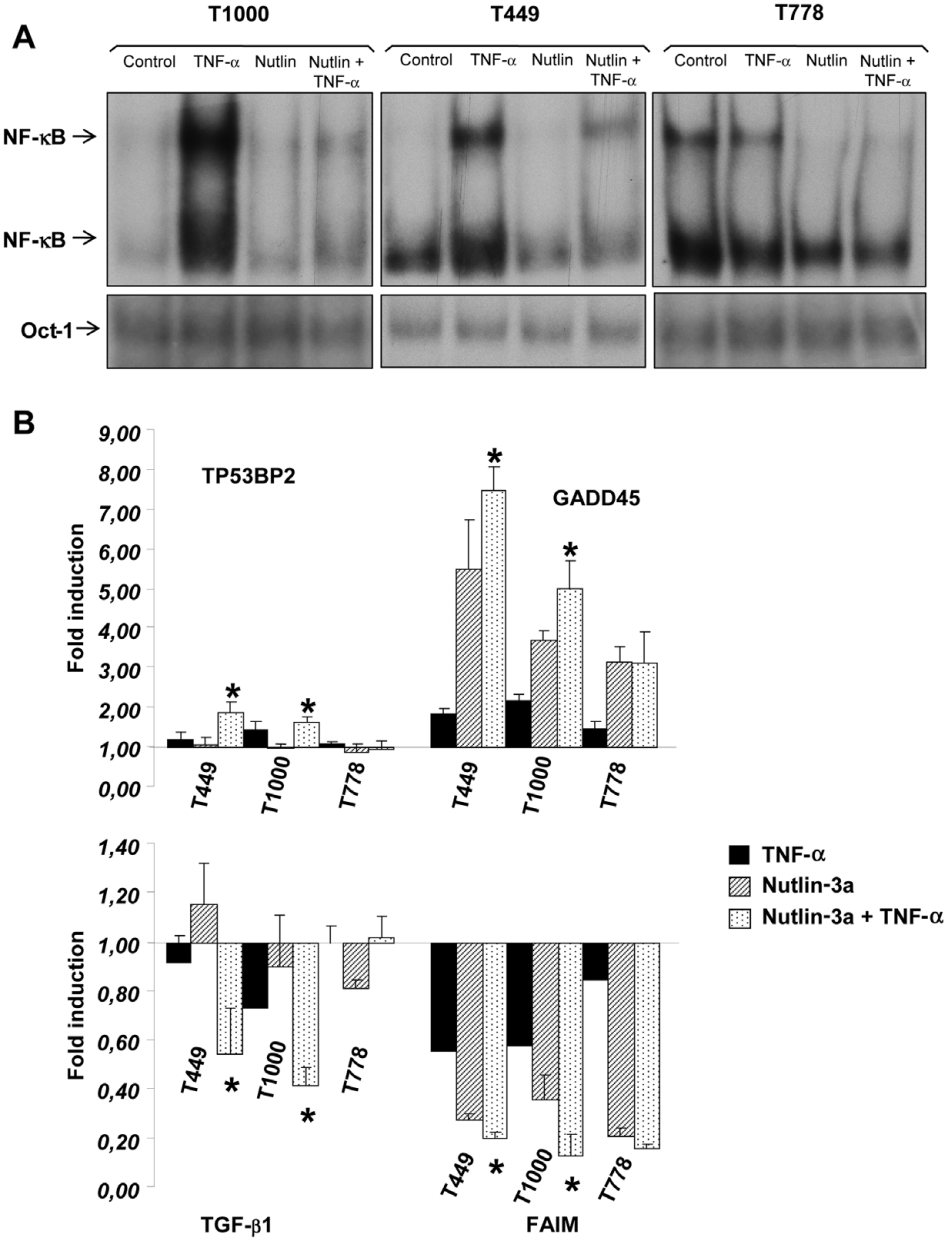
Differential NF-κB-DNA-binding and apoptotic gene expression are involved in the potentiating effect of TNF-α and Nutlin-3a treatment. A, NF-κB DNA binding activity after treatment of the TP53Wt/MDM2Ampl cell lines with TNF-α or/and Nutlin-3a. Nuclear extracts from monolayers of T449, T778 and T1000 cells, untreated or treated with either TNF-α, or Nutlin-3a or both for 72 h, were analyzed for NF-κB activity by EMSA. B, Differentially regulated apoptotic genes in TP53Wt/MDM2Ampl cell lines treated with the combination of TNF-α and Nutlin-3a compared to Nutlin-3a-only treated cells. T449, T778 and T1000 cell lines were treated 16 h with 50 ng/ml TNF-α and/or 5 µmol Nutlin-3a. Total RNA was extracted and analyzed by qRT-PCR. Results are expressed as mean ± SD from two independent experiments performed in duplicate. * p<0.05 in TNF-α + Nutlin-3a treated cells as compared to Nutlin-3a alone.

It is well established that NF-κB plays a major role in TNF-α-mediated survival [38]. Moreover, the relationship between p53, TNF-α and NF-κB has been analyzed in MCF-7 breast cancer cells. In this model, TNF-α was able to induce transcription of p21 and accumulate inactive p53, but inhibition of the NF-κB pathway abrogated p21 transcription [39]. Therefore, we investigated whether combination of Nutlin-3a and TNF-α in sarcoma cells interferes with the NF-κB-activity, which might explain the observed potentiation of TNF-α-induced cell death in the presence of Nutlin-3a. For this purpose, we analyzed NF-κB-DNA binding activity by Electrophoretic Mobility Shift Assay (EMSA) in T1000, T449 and T778 cells. TNF-α treatment induced NF-κB binding activity in T1000 and T449 compared to that seen in untreated cells or cells treated with Nutlin-3a alone. Importantly, NF-κB DNA binding was markedly decreased when Nutlin-3a and TNF-α were combined (Fig. 6A). Remarkably, in the T778 cell line, significant constitutive binding of NF-κB to DNA was observed, which was inhibited in the presence of Nutlin-3a. This constitutive binding may explain the particular sensitivity of this cell line to Nutlin-3a and thus the absence of amplification of the effect with TNF-α. Furthermore, in order to understand the mechanisms involved in the attenuation of cell lines resistance to TNF-α, we analysed the expression level of 94 genes potentially involved in the regulation of apoptosis in T449 and T1000 versus T778 cells using real-time PCR. No significant difference was observed in the expression of most genes tested between these two types of cell lines (data not shown). However in T449 and T1000 cells, TNF-α combined with Nutlin-3a significantly increased the mRNA levels of TP53BP2 and GADD45, which are involved in the inhibition of cell-cycle progression and apoptosis promotion (Fig. 6B). TNF-α combined with Nutlin-3a also significantly decreased the mRNA levels of the anti-apoptotic genes TGF-β1 and FAIM. The mRNA levels of all these genes were unchanged in the T778 cell line.

## Discussion

TNF-α combined with melphalan has become the standard treatment for advanced limb sarcoma with a good benefit/risk ratio [4] and a remarkable response rate in human patients with heavily pre-treated disease. Clinical information gained from radiological studies and pathological biopsies showed that TNF-α targeted the tumour vasculature and had no visible direct effects on tumour cells [40], although direct selective cytotoxic activity against tumour cells has been described *in vitro* [17] with wide variation of tumour cell sensitivity. In the course of these studies, we asked whether in the context of ILP, tumour cell killing could also play a role in addition to the vascular effect of TNF-α. Our data indicate that TNF-α was able to induce *ex vivo* apoptosis of sarcoma tumour cells.

It should be noted that one of the hallmarks of STS is their pronounced resistance to apoptosis, resulting in cell survival or absence of cell damage even when confronted to multiple stress stimuli [2]. Indeed, in our *in situ* cell death detection experiments, two out of eight STS tumours appeared to be completely resistant to TNF-α effects and three others displayed partial tumour resistance. Accumulating evidence indicates that p53 dysregulation is very common in STS and that p53 mutations correlate with increased resistance to current therapeutic strategies [23]. Since a relationship between *TP53* status and tumour sensitivity to TNF-α was previously reported in a human breast cancer model [18], [19], and given that drugs restoring p53 function are now available, we investigated the impact of p53 in the context of resistance to TNF-α in STS cell lines. We demonstrated that STS tumour resistance to TNF-α-induced-cell death was also associated with p53 inactivation (by point mutation or MDM2 amplification). Moreover, p53 silencing in a *TP53^Wt^* cell line inhibited sensitivity to TNF-α-cytotoxicity, and p53 overexpression in *TP53^Null^* cells restored it. These data strengthen the critical role of p53 in TNF-α-induced cell death in STS. However, a role for p53-independent pathways such as those related to p73/63 [41] have been reported to induce apoptosis. Since our p53-inactivated cell lines were not consistently killed by TNF-α, these alternate pathways are not obviously involved in the response to TNF-α in our STS cell lines.

It is well known that the conformation of the DNA-binding domain of p53 is flexible and that conformational changes in mutant p53 are reversible. Thus, restoration of p53 wild-type function to the highly accumulated mutant p53 in tumour cells may possibly result in a considerable therapeutic response [24]. In this regard, we investigated the effect of CP-31398, a small molecule known to rescue p53 functions. In this report, we provide evidence that this compound was able to attenuate STS resistance to TNF-α. An improvement of TNF-α killing efficiency can thus be obtained when used in association with p53 re-activating agents in the context of p53 mutation.

Moreover, the recent use of MDM2 inhibitors of the Nutlin group has been reported to be a promising new therapeutic strategy for human tumours retaining wild-type p53 status. Nutlin-3a is a highly selective inhibitor of the MDM2-p53 interaction by competitive binding. Consequently, it potently stabilizes and activates wild-type, but not mutant p53 protein in tumour cells in contrast to chemotherapeutics whose mechanism of action, at least in part, relies on genotoxic activation of p53. Importantly, there is evidence that Nutlins, while being toxic to cancer cells, do not induce cell death or apoptosis in normal non-malignant cells and tissues [27], [42]. In our study, Nutlin-3a effectively restored the p53 pathway in the *TP53^Wt^*/*MDM2^Ampl^* liposarcoma cell lines tested. More importantly, the combination of Nutlin-3a and TNF-α could reverse the observed resistance of *TP53^Wt^*/*MDM2^Ampl^* sarcoma cell lines to TNF-α.

It has been reported that in p53-mutated cell lines, NF-κB was induced at higher levels following TNF-α treatment [43] and that TNF-α-induced apoptosis in these cells was modulated by mutated p53 as well as by the NF-κB induction [44]. Moreover, it is well known that NF-κB has a protective action against TNF-α-induced cell death, suggesting that NF-κB inhibition may potentiate TNF-α-induced apoptosis [45]. In this regard, Nutlin-3a was shown to suppress TNF-α-induced NF-κB reporter activation, and to inhibit transactivation of NF-κB target genes [46]. Here we show that TNF-α-induced NF-κB-DNA binding is differentially modulated in *TP53^Wt^*/*MDM2^Ampl^* sarcoma cell lines, demonstrating a sensitization effect with the combined treatment of TNF-α and Nutlin-3a. In addition, our data highlight that some pro-(TP53BP2 and GADD45) and anti-apoptotic genes (TGF-β1 and FAIM) display a different pattern of transcript induction and repression in sensitized cell lines when the combination of Nutlin-3a and TNF-α is used. Further studies are thus needed to determine the molecular mechanisms associated with the potentiating pro-apoptotic effect of TNF-α combined with Nutlin-3a, and to determine whether NF-κB-DNA binding repression by Nutlin-3a is involved in the observed potentiation of apoptosis when both TNF-α and Nutlin-3a are used. Indeed, this may have important implications since the use of both CP-31398 and Nutlin-3a, which act on various pathways, may be useful in overcoming drug resistance and thus could result in a more efficient therapeutic response.

In conclusion, our data indicate that activating p53 with CP-31398 or Nutlin-3a, in respectively *TP53^Mut^* and *TP53^Wt^*/*MDM2^Ampl^* cell lines, restores sensitivity to TNF-α. This is of importance in the clinical setting where the use of TNF-α administered locally and in combination with other drugs that restore the p53 pathway may contribute to improved efficacy of such therapy. These findings thus provide a new strategy for treating highly refractory STS, in which p53 is frequently mutated or inactivated.
